# The Role of Berberine in the Prevention of HIF-1*α* Activation to Alleviate Adipose Tissue Fibrosis in High-Fat-Diet-Induced Obese Mice

**DOI:** 10.1155/2018/4395137

**Published:** 2018-12-02

**Authors:** Meilin Hu, Fan Wu, Jinlong Luo, Jing Gong, Ke Fang, Xueping Yang, Jingbin Li, Guang Chen, Fuer Lu

**Affiliations:** ^1^Institute of Integrated Traditional Chinese and Western Medicine, Tongji Hospital, Tongji Medical College, Huazhong University of Science and Technology, Wuhan 430030, China; ^2^Department of Emergency, Tongji Hospital, Tongji Medical College, Huazhong University of Science and Technology, Wuhan 430030, China; ^3^Department of Integrated Traditional Chinese and Western Medicine, Tongji Hospital, Tongji Medical College, Huazhong University of Science and Technology, Wuhan 430030, China

## Abstract

Berberine (BBR) is the main active ingredient of a traditional Chinese herb* Coptis chinensis*. It has been reported to exhibit beneficial effects in treating diabetes and obesity. However, the underlying mechanism has not been fully elucidated. Adipose tissue fibrosis is a hallmark of obesity-associated adipose tissue dysfunction. HIF-1*α* plays a key role in adipose tissue fibrosis, which closely linked to metabolic dysfunction in obese state. We hypothesized that BBR may alleviate obesity-induced adipose tissue fibrosis and associated metabolic dysfunction through inhibition of HIF-1*α*. To test this hypothesis, we treated high fat diet (HFD) feeding mice with different dose of BBR (100 mg/kg, 200 mg/kg, and 300 mg/kg) for 8 weeks. We found that BBR treatment greatly decreased the body weight gain and reduced insulin resistance induced by HFD. Data also revealed that BBR improved histologic fibrous of epididymal white adipose tissue (eWAT) and was accompanied with inhibition of the abnormal synthesis and deposition of extracellular matrix (ECM) proteins, such as collagen and fibronectin. We also found that BBR treatment suppressed the expression of HIF-1*α* and decreased the mRNA expression of LOX in epididymal adipose tissue, which plays a key role in fibrosis development. Taken together, these results suggest that BBR can regulate metabolic homeostasis and suppress adipose tissue fibrosis through inhibiting the expression of HIF-1*α*.

## 1. Introduction

Obesity has become a severe health issue and causes enormous economic burden worldwide. A study from 195 countries worldwide showed that the prevalence of obesity has doubled since 1980. The obesity rates for children and adults were 5% and 12%, respectively, and these results are similar with the prevalence of type 2 diabetes worldwide [1]. Obese individuals are more likely to develop metabolic disorders, such as type 2 diabetes and cardiovascular diseases, as well as chronic inflammatory diseases [2]. Dietary and life style changes are recommended for obese people. However, it only works for a small percent of people. A better understanding of the pathological changes of obesity would provide potential new target for antiobese therapy.

It is generally accepted that oxygen supply fails to meet the demand of expanding adipose during the progression from the lean to the obese state, leading to relative hypoxia in adipose tissue. Moreover, it has been reported that, early in the course of high-fat diet feeding, the consumption of oxygen is increased [3]. Therefore hypoxia is an early event in adipose tissue dysfunction. Consequently, hypoxic conditions trigger hypoxia-inducible factor 1*α* (HIF-1*α*) expression. Unlike its role in tumor, HIF-1*α* fails to induce proangiogenic response by using a transgenic model of overexpression of HIF-1*α*. Instead, HIF-1*α* induces an alternative transcriptional program, resulting in enhanced synthesis of ECM components and eventually promoting the development of white adipose tissue fibrosis [4]. Adipose tissue fibrosis is increasingly accepted as a hallmark of obesity-associated adipose tissue dysfunction which is usually characterized by an excessive accumulation of ECM proteins [5]. ECM consists of various components such as collagens and fibronectins. At the early stage of obesity, ECM on WAT not only provides supportive scaffolds for the tissues but also is involved in many physiological and pathological processes. Sustaining high flexibility of ECM ensures the healthy expansion of WAT. However, with the development of obesity, excessive ECM deposition leads to fibrosis which compromises the function of adipose tissue and then leads to obesity-associated metabolic disorders [6]. Fibrosis in WAT reduces ECM flexibility and tissue plasticity, causing activation of inflammatory signals and ectopic deposition of lipids in other tissues [7]. The outcome of ECM remodeling in WAT has been reported in mice. In db/db mice, the mRNA levels of collagen I, IV, and VI are overexpressed in WAT. With the deletion of collagen VI, the mice display increased adipocyte size and decreased adipocyte necrosis and improvement of insulin sensitivity [8]. A similar result has been reported in obese individuals who display increased inflammatory macrophages infiltration and elevated extent of fibrosis, as well as the abnormal deposition of ECM in WAT [9]. Changes in ECM and subsequent fibrosis in WAT are closely associated with insulin resistance and the development of type 2 diabetes [10]. Thus treatment of fibrosis has been considered as an important and effective strategy to postpone the development of obesity and diabetes associated metabolic disorders.

Berberine (BBR) is categorized as isoquinolinate alkaloids. It exists in plants such as* Coptis chinensis* and* Phellodendron amurense*. Its pharmacological activities consist of anti-inflammatory [11], lowering blood glucose, and reducing lipid accumulation [12]. The antifibrosis effect of BBR has been well investigated in kidney and liver [13, 14]. However, its effect on adipose tissue is still obscure, especially at the state of hypoxia. It has been found that BBR is able to suppress HIF-1*α* which is correlated with its anticancer effect [15]. The expanding adipose tissue and tumor share the similar hypoxic microenvironment. Thus, we investigated the effect of BBR on the regulation of HIF-1*α* activation in adipose tissue with focus on fibrosis. In the current study, we found that mice treated with BBR show a beneficial metabolically phenotype, such as a decrease in body weight gain and reduced insulin resistance, which was accompanied by decreased HIF-1*α* accumulation and improved adipose tissue fibrosis.

## 2. Materials and Methods

### 2.1. Animals

All animal protocols were reviewed and approved by the Animal Ethics Committee of Tongji Medical College, Huazhong University of Science and Technology, China. Seven-week-old Specific Pathogen Free (SPF) male C57BL/6 mice were purchased from Hubei Provincial Center for Disease Control and Prevention. Mice were kept in environmentally controlled room (20±2°C, 60±5% humidity, and 14/10 circadian rhythm), with water and diets were provided* ad libitum*. After one-week acclimation, mice were fed with normal chow diet (NC) or high-fat-diet (HFD, # MD12033, 60% calories from fat; Research Diet, Mediscience Ltd, China) for 16 weeks. Then mice were randomly divided into four groups and gavaged daily with either vehicle (0.9% saline) or different concentrations of BBR suspension (Sigma, 100, 200, and 300 mg/kg) for another 8 weeks. Body weight and food intake were measured weekly.

### 2.2. Glucose Tolerance Test (GTT) and Insulin Tolerance Test (ITT)

For glucose tolerance test, mice were fasted overnight and injected with glucose intraperitoneally (i.p., 1.5 g/kg body weight). Blood glucose concentrations were measured at 0, 30, 60, and 120 min after glucose injection from the tail with glucose strip (ACCU-CHEK Performa, Roche). For insulin tolerance test, mice were fasted for 6 hours and injected with 0.75 U/kg insulin (i.p.). Blood glucose concentrations were measured at 0, 30, 60, and 120 min after injection [16].

### 2.3. Western Blot

Epididymal white adipose tissues were lysed in RIPA buffer. Twenty to thirty microgram protein samples were separated on 10-12% SDS-PAGE and transferred onto 0.45* μ*m nitrocellulose membranes. Primary antibodies against *α*-SMA (Abcam, UK), collagen I (Abcam, UK), Fibronectin (Invitrogen, USA), PDGFR-*α* (Santa Cruz, USA), HIF-1*α* (Cell Signaling, USA), and cleaved caspase 3 (Cell Signaling, USA) were incubated with the membrane overnight at 4°C. After incubation of fluorescence-labeled secondary antibodies, bands were visualized using near-infrared fluorescence imaging system (Odyssey, NE, USA).

### 2.4. Quantitative Real-Time Polymerase Chain Reaction (RT-qPCR)

Total RNA was extracted from epididymal white adipose tissue using Trizol Reagent (TaKaRa, Japan). After reverse-transcription, RT-qPCR was performed using StepOne™ Real-Time PCR System (StepOne, USA) and analyzed by 2^−ΔΔCT^ method with *β*-actin as internal control. The primer sequences are shown in Table 1.

### 2.5. Histological and Immunohistochemistry (IHC) Staining

Fresh collected epididymal white adipose tissues were fixed in 10% neutral buffered formalin solution. Hematoxylin & Eosin (H&E) staining was applied to determine the morphology and size of adipocytes. More than 200 cells were counted from 4 mice in each group. Cell size was calculated by Image Pro Plus. Collagen deposition was determined by Masson trichrome and Sirius Red staining. For IHC, tissue slides were dewaxed by dimethylbenzene, polarized with descending concentrations of alcohol, and rinsed with deionized water. Antigen was retrieved in 10 mM citric acid (pH 6.0) with indicated microwave setting. Slides were incubated in 3% hydrogen peroxide for 30 min to block endogenous peroxidase activity. The 10% goat serum was used to block the slides for 60 min. Primary antibodies were applied overnight at 4°C followed by incubation of HRP-conjugated secondary antibody for 60 min at room temperature. Staining was visualized by DAB and counterstaining was performed with hematoxylin.

### 2.6. Statistical Analysis

Data were presented as mean ± SEM and analyzed using SPSS 17.0 software. One-way analysis of variance (ANOVA) was performed with* p*<0.05 being considered of statistical significance.

## 3. Results

### 3.1. BBR Decreases the Body Weight Gain and Glucose Level and Reduces Insulin Resistance in HFD Mice

**Figure 1:**
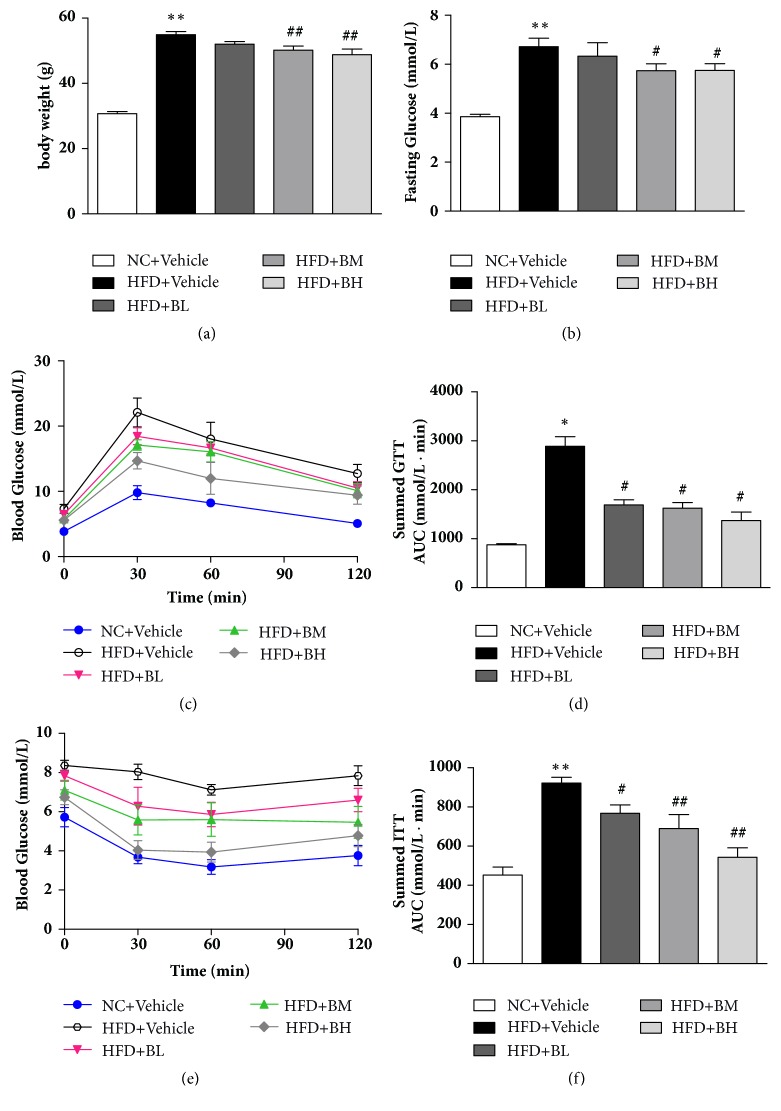
**Protective effects of berberine on HFD-induced obesity.** Male C57BL/6 mice were fed a high-fat-diet (HFD) or normal chow diet (NC) for 16 weeks and then given BBR for 8 weeks (100mg/kg/day, BL; 200mg/kg/day, BM; and 300mg/kg/day, BH) or saline (Vehicle). Body weight (BW) was measured (n=6) (a). Fasting glucose levels quantified in overnight-fasted mice (b). Blood glucose was measured during glucose tolerance test (GTT) in overnight fasted mice (n=5) (c). Areas under the curve (AUC) were measured for GTT (d). Blood glucose was measured during insulin tolerance test (ITT) in 6-hour-fasted mice (n=5) (e). Areas under the curve (AUC) were measured for ITT (f). *∗*P < 0.05 versus NC group, *∗∗*P < 0.01 versus NC group; ^#^P < 0.05 versus HFD group; ^##^P < 0.01 versus HFD group.

To test whether BBR feeding can prevent the HFD-induced metabolic dysfunctions, 7-week-old male C57BL/6J mice were fed with HFD for 16 weeks and then given BBR for another 8 weeks. BBR feeding significantly decreased the body weight gain compared to control mice (Figure 1(a)). HFD significantly increased fasting glucose level and BBR treatment greatly reduced this HFD-induced glucose increase (Figure 1(b)). Moreover, glucose tolerance test and insulin tolerance test indicated that BBR treated mice have increased glucose clearance rate (Figure 1(c)) and improved insulin sensitivity (Figure 1(e)). We also calculated the area under the curve for GTT (Figure 1(d)) and ITT (Figure 1(f)), which clearly showed that BBR treatment significantly improved glucose tolerance and insulin sensitivity.

### 3.2. BBR Inhibits Adipose Tissue Fibrosis in HFD Mice

**Figure 2:**
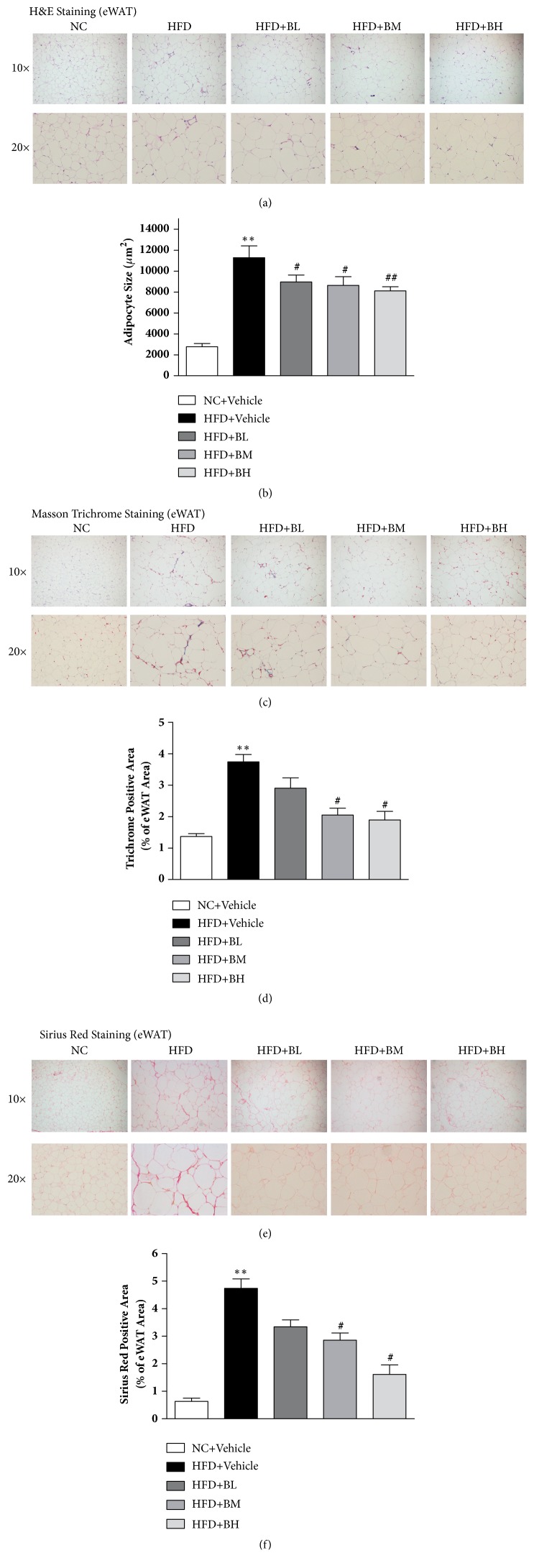
**Effects of berberine on eWAT fibrosis of HFD-induced obese mice. **Histological analysis of H&E staining of epididymal fat sections. Adipocyte area was analyzed by Image Pro Plus software ((a) and (b)). The deposition of collagen in epididymal white adipose tissue was determined by Masson trichrome staining (c, d) and Sirius Red staining (e, f). Data are shown as mean ± SEM (n=4). *∗∗*P < 0.01 versus NC group; ^#^P < 0.05 versus HFD group; ^##^P < 0.01 versus HFD group.

HFD feeding significantly increased the body weight and the cell size of epididymal fat tissue compared to normal chow feeding, while BBR treatment greatly decreased the cell size of epididymal fat tissue (Figures 2(a) and 2(b)). Masson trichrome staining revealed that HFD feeding induced clusters of adipocytes that were segmented by bundle- and rope-like collagens, which is a hall marker of fibrosis. Such bundle- and rope-like collagens staining was significantly decreased by BBR treatment (Figures 2(c) and 2(d)). Sirius Red staining of epididymal fat tissue reviewed similar decreased levels of collagen deposition in BBR treated mice (Figures 2(e) and 2(f)).

### 3.3. BBR Inhibits the Abnormal Synthesis of ECM Protein in HFD Mice

**Figure 3:**
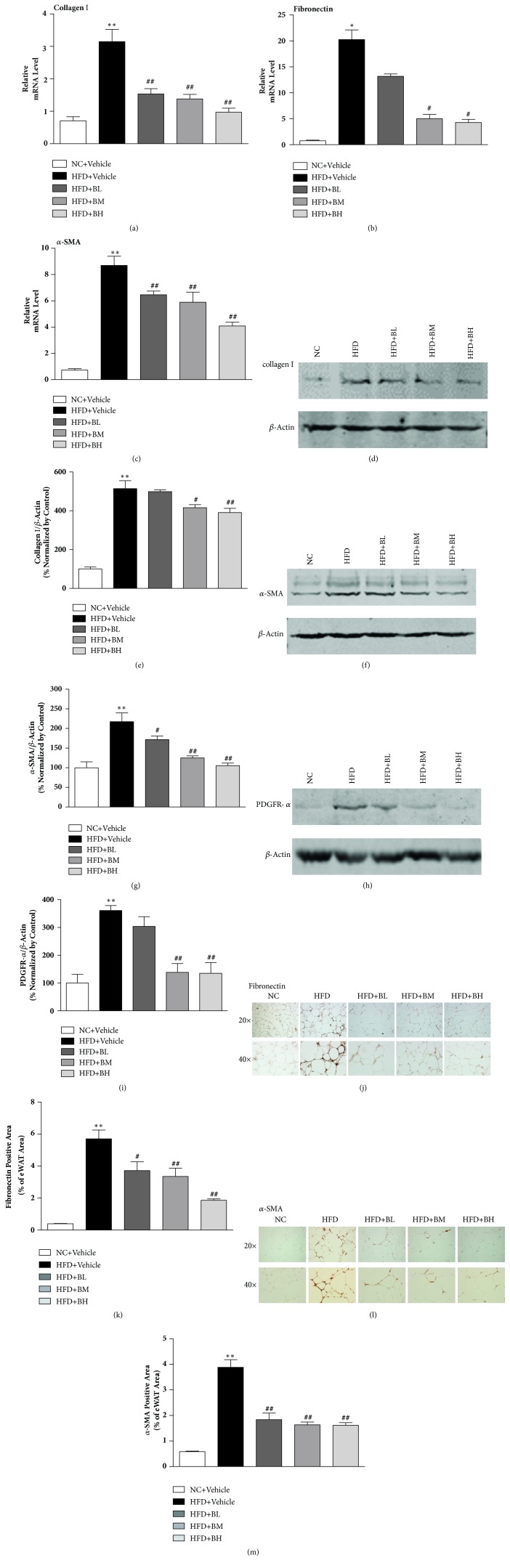
**Berberine attenuated ECM components in eWAT of HFD-induced obese mice. **RT-PCR analysis for relative mRNA expression of collagen-I, Fibronectin, *α*-SMA in the eWAT of normal chow- and HFD-fed mice (n=6) (a-c). Western blot analysis of collagen-I, *α*-SMA, and PDGFR-*α* in the eWAT of chow- and HFD-fed mice (n=3) (d-i). Immunohistochemistry and quantitative analysis for Fibronectin in the eWAT of chow- and HFD-fed mice (n=3) ((j) and (k)). Immunohistochemistry and quantitative analysis for *α*-SMA in the eWAT of chow- and HFD-fed mice (n=3) ((l) and (m)). Data are shown as mean ± SEM. *∗*P < 0.05 versus NC group; *∗∗*P < 0.01 versus NC group; ^#^P < 0.05 versus HFD group; ^##^P < 0.01 versus HFD group.

The decreased deposition of collagen by BBR treatment may be through the regulation of synthesis and secretion of ECM proteins. RT-qPCR results showed that HFD significantly enhanced collagen I transcription in eWAT. However, BBR reversed such activation (Figure 3(a)). This was further supported by Western blot analysis (Figures 3(d) and 3(e)). HFD-induced activation on fibronectin synthesis, another fibrosis-related ECM protein, was also found to be attenuated by BBR at both mRNA (Figure 3(b)) and protein (Figures 3(j) and 3(k)) levels. Moreover, we also found that HFD feeding dramatically induced the expression of smooth muscle actin (*α*-SMA) (Figures 3(c), 3(f), 3(g), 3(l), and 3(m)) and platelet-derived growth factor receptor alpha (PDGFR-*α*) (Figures 3(h) and 3(i)) and these increases were attenuated by BBR treatment. Taken together, our data suggests that BBR treatment attenuates HFD-induced fibrosis and fibroblast proliferation.

### 3.4. BBR Inhibits HIF-1*α* and Lysyl Oxidase (LOX) Expression in HFD Mice

**Figure 4:**
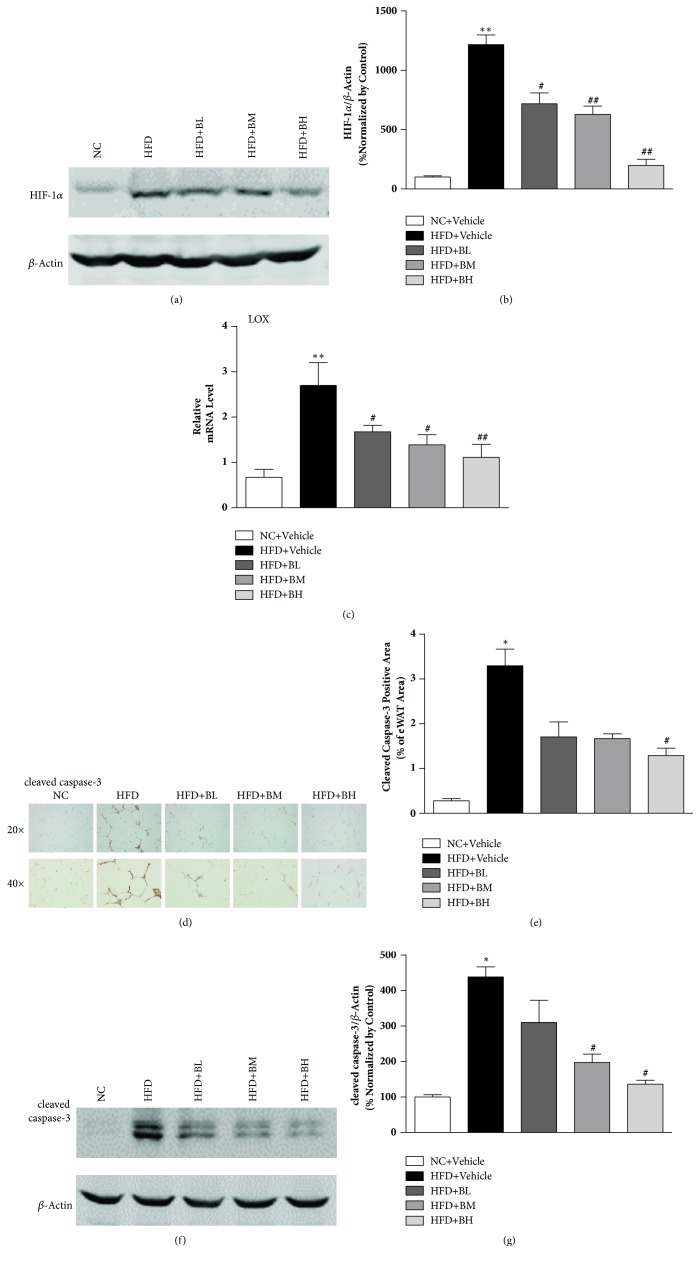
**Berberine suppressed HIF-1** **α** **and adipocyte apoptosis in eWAT of HFD-induced obese mice. **Western blot analysis for HIF-1*α* expression (n=3) (a, b); RT-qPCR analysis for relative mRNA expression of LOX (n=6) (c). Immunohistochemistry and quantitative analysis for cleaved caspase-3 (n=3) (d, e). Western blot analysis for cleaved caspase-3 expression (n=3) (f, g). Data are shown as mean ± SEM. *∗*P < 0.05 versus NC group; *∗∗*P < 0.01 versus NC group; #P < 0.05 versus HFD group; ##P < 0.01 versus HFD group.

Previous studies found that activation of HIF-1*α* will initiate WAT fibrosis and insulin resistance [17]. To test whether BBR reverse HFD-induced adipose tissue fibrosis through above pathway, we analyzed the HIF-1*α* levels in the eWAT of HFD fed mice or BBR treated mice. As shown in Figures 4(a) and 4(b), HFD feeding activated HIF-1*α*. Strikingly, BBR treatment greatly reversed those HFD feeding induced activation of HIF-1*α*. Moreover, LOX, the transcriptional target of HIF-1*α*, was also decreased by BBR (Figure 4(c)). Interestingly, we also found that BBR treatment attenuated the HFD-induced expression of cleaved caspase-3 (Figures 4(d), 4(e), 4(f), and 4(g)) in WAT, which suggested that BBR treatment also prevented the HFD-induced adipocyte apoptosis.

## 4. Discussion

Fibrosis is a common pathological process in which abnormal ECM are produced and may lead to targeted organ failure at the end stage. Like many other fibrotic diseases, adipose tissue fibrosis occurs during the development of obesity and causes systematic metabolic dysregulation. Antifibrotic therapies are crucial in preventing disease progression. Our study suggests that BBR treatment can suppress abnormal extracellular matrix remodeling in adipose tissue and modulate the metabolic homeostasis. In this study, 7-week-old male C57BL/6 mice were fed with HFD for 16 weeks and treated with BBR for another 8 weeks. Consistent with previous reports [18], BBR significantly reduced HFD-induced body weight gain. Mouse glucose metabolism in terms of blood glucose clearance and insulin sensitivity was also improved by BBR treatment in a dose dependent manner (100 to 300 mg/kg). Previous studies have found that BBR is effective and safe in treating obese rodents and patients [19, 20]. We found that BBR alleviated WAT fibrosis through decreasing adipocyte abnormality and collagen deposition. Those antifibrosis roles of BBR were also found in mouse models with renal or liver fibrosis by other researchers [13, 14].

Identification of ECM components is essential to have a better understanding of adipose tissue fibrosis. Generally, fibroblasts are portrayed for its expression of *α*-SMA and secretion of collagens. It plays a key role in tissue repairing. However, excessive synthesis of collagen on fibroblast is closely associated with fibrosis [21]. Although the cell origin for adipose tissue fibrosis has not been fully illustrated, adipose progenitor cell (APC) is considered as the major cells for the ECM synthesis in adipose tissue. The induction of fibroblast is associated with the expression of *α*-SMA on APC. Activation of PDGFR-*α* on APC drives the cell differentiating and promotes fibrosis [22]. Interestingly, HFD-induced activation of *α*-SMA and PDGFR-*α* was inhibited by BBR in our study. These results indicate that BBR could suppress the abnormal ECM remodeling in adipose tissue and slow down the development of adipose tissue fibrosis in obese mice induced by HFD. Consistent with previous study that excessive synthesized ECM triggered adipocyte necrosis [3], in obese state, the adipose tissue is relative hypoxia.

HIF-1*α* is the major mediator of hypoxia response. Instead of activating the angiogenic programme, HIF-1*α* activates potent profibrotic transcriptional program that increases ECM protein expression, resulting in adipose tissue fibrosis [4]. The important role of HIF-1*α* in the progression of insulin resistance has been well documented. Activation of HIF-1*α* in adipose tissue leads to insulin resistance [23], while silence of HIF-1*α* in adipocytes protects mice from glucose tolerance by enhancing insulin secretion [24]. Previous research indicated that HIF-1*α* plays a key role in the development of organ fibrosis, such as lung, kidney, and adipose tissue [25, 26]. It was reported that inhibition of HIF-1*α* improves adipose tissue fibrosis and attenuates metabolic inflammation in the obese mice induced by high-fat diet, which suggests HIF-1*α* is closely related to the adipose tissue fibrosis [27]. In current study, we found that HFD induced the upregulation of HIF-1*α* protein in eWAT, and BBR can significantly suppress the upregulation of HIF-1*α*. This result indicates that BBR could ameliorate hypoxia in the eWAT. Inhibition of HIF-1*α* may be involved in the antifibrotic effect of BBR. As the target of HIF-1*α*, LOX is a crucial factor in collagen fiber formation, which promotes the cross-linking of collagen. Inhibition of LOX activity in transgenic HIF-1*α* mice resulted in an improvement in the insulin sensitivity which highlights the critical role of LOX in HIF-1*α* mediated fibrosis and insulin resistance [24]. Our research showed that BBR can decrease the mRNA expression of LOX in eWAT at the state of obesity induced by HFD. This indicates that BBR could suppress the collagen fiber formation through inhibiting the LOX expression. Adipocyte death is prevailing under local hypoxia [28], as well as a major driver for the adipose tissue remodeling [29]. Our current study found that cleaved caspase-3 characterized by cell apoptosis and necrosis was increased in adipocytes from HFD mice. However, BBR greatly reduce the level of cleaved caspase-3, which suggests that BBR treatment can prevent HFD-induced adipocytes apoptosis. As we all know, hypoxia microenvironment is early event in the development of adipose tissue fibrosis. In this study, we can conclude that BBR could inhibit the adipose tissue fibrosis by improving the hypoxia microenvironment of eWAT.

In conclusion, BBR can restore metabolic homeostasis and remarkably alleviates HFD-induced adipocyte apoptosis in white adipose tissues. The data in this study indicates that BBR suppresses adipose tissue fibrosis and its abnormal ECM remodeling. The inhibition of HIF-1*α* may play a critical role in the antifibrotic effect of BBR. These results provide new insight into developing BBR based compounds for treating obesity-associated metabolic disorders.

## Figures and Tables

**Table 1:** Primer sequence.

	** Forward **	** Reverse**
*β*-Actin	CTGAGAGGGAAATCGTGCGT	CCACAGGATTCCATACCCAAGA
Collagen I	CAAGAAGACATCCCTGAAGTC	ACAGTCCAGTTCTTCATTGC
*α*-SMA	CCCAGACATCAGGGAGTAATGG	TCTATCGGATACTTCAGCGTCA
LOX	GCTGCACAATTTCACCGTATTA	TGTCATTTGTTGCGAAAGAGTCC
Fibronectin	GAAATGGAAAAGGGGAATGG	CGTTGCATCTGTTTCTGGAGGT

## Data Availability

The data used to support the findings of this study are included within the article.
